# Supplementary material for: Transferrin receptor 1 (TfR1) functions as an entry receptor for scale drop disease virus to invade the host cell via clathrin-mediated endocytosis
Source: J Virol. 2025 Jul 28;99(8):e00671-25. doi: 10.1128/jvi.00671-25 (PMC12363161; doi:10.1128/jvi.00671-25)
Supplement: Table S1 — Identification of TfR1 in purified SDDV virions by LC-MS/MS analysis. [file jvi.00671-25-s0001.docx]

**Table 1** Identification of TfR1 in purified SDDV virions by LC-MS/MS analysis

|  | Reference | PepCount | UniquePep  Count | Cover  Percent | MW | PI |
| --- | --- | --- | --- | --- | --- | --- |
| $11-1 | QEU52702.1 transferrin receptor  protein 1 [*Siniperca chuatsi*] | 5 | 5 | 6.49% | 84703.38 | 7.69 |
