# Supplementary material for: Transferrin receptor 1 (TfR1) functions as an entry receptor for scale drop disease virus to invade the host cell via clathrin-mediated endocytosis
Source: J Virol. 2025 Jul 28;99(8):e00671-25. doi: 10.1128/jvi.00671-25 (PMC12363161; doi:10.1128/jvi.00671-25)
Supplement: Supplemental table legends [file jvi.00671-25-s0002.docx]

**Supporting Information**

**Table S1** Identification of TfR1 in purified SDDV virions by LC-MS/MS analysis.

**Table S2** Primers used for plasmid construction.

**Table S3** Primers used for RT-Qpcr.
