# Supplementary material for: Transferrin receptor 1 (TfR1) functions as an entry receptor for scale drop disease virus to invade the host cell via clathrin-mediated endocytosis
Source: J Virol. 2025 Jul 28;99(8):e00671-25. doi: 10.1128/jvi.00671-25 (PMC12363161; doi:10.1128/jvi.00671-25)
Supplement: Table S2 — Primers used for plasmid construction. [file jvi.00671-25-s0003.docx]

**Table 2** Primers used for plasmid construction

| Primer | Primer sequence (5’-3’) | Vector |
| --- | --- | --- |
| Pld1-GFP-F | tcgagctcaagcttcgaattcTGCCACCATGAGTGAGTTTTCCAGTGACAGCCA | pEGFP-N3 |
| Pld1-GFP-R | cgggcccgcggtaccgtcgacGGGGTCCTGAACCTTAACAAAGT |  |
| Ad-GFP-F | tcgagctcaagcttcgaattcTGCCACCATGCCAGCTTCTGGCTCCAACAG |  |
| Ad-GFP-R | cgggcccgcggtaccgtcgacGTTGACCTCCACGGTAATAATATTGC |  |
| Pld2-GFP-F | tcgagctcaagcttcgaattcTGCCACCATGAACGTTCCCACTGAGAAGAAGATAA |  |
| Pld2-GFP-R | cgggcccgcggtaccgtcgacGGTGTCCAGCATTGTGCCA |  |
| Fl-GFP-F | tcgagctcaagcttcgaattcTGCCACCATGCGGGAGAAACTGAACGCGG |  |
| Fl-GFP-R | cgggcccgcggtaccgtcgacGATTTCATTATCCAAAGACCAGATGT |  |
| Remove-GFP-F | tcgagctcaagcttcgaattcATGGACCAGGCAAGGTCAACG |  |
| Remove-GFP-R | cgggcccgcggtaccgtcgacCTCGATGTCCTCCAGGTTGC |  |
| MCP-Flag-F | tcgagctcaagcttcgaattcATGTCATCTATTGCAGGAGCTAATG | pFlag-N3 |
| MCP-Flag-R | cgggcccgcggtaccgtcgacTTACAAGATCGGAAATCCAAATGA |  |
