# Supplementary material for: Transferrin receptor 1 (TfR1) functions as an entry receptor for scale drop disease virus to invade the host cell via clathrin-mediated endocytosis
Source: J Virol. 2025 Jul 28;99(8):e00671-25. doi: 10.1128/jvi.00671-25 (PMC12363161; doi:10.1128/jvi.00671-25)
Supplement: Table S3 — Primers used for RT-qPCR. [file jvi.00671-25-s0004.docx]

**Table 3** Primers used for qRT-PCR

| Primer | Primer sequence (5’-3’) |
| --- | --- |
| β-actin-F | AGAGGGAAATCGTGCGTG |
| β-actin-R | GAAGGAAGGCTGGAAGAGG |
| SDDV-F | AAGAGCGTGAAGCAATGTC |
| SDDV-R | GGGATGACTAAATCGCAGA |
| TfR1-F | TCCCTAAACAAGCCTGCGACTC |
| TfR1-R | CGCTATGTGACCTGCGAACC |
